# Dietary sources and intakes of folates and vitamin B_12_ in the Spanish population: Findings from the ANIBES study

**DOI:** 10.1371/journal.pone.0189230

**Published:** 2017-12-15

**Authors:** Teresa Partearroyo, María de Lourdes Samaniego-Vaesken, Emma Ruiz, Josune Olza, Javier Aranceta-Bartrina, Ángel Gil, Marcela González-Gross, Rosa M. Ortega, Lluis Serra-Majem, Gregorio Varela-Moreiras

**Affiliations:** 1 Department of Pharmaceutical and Health Sciences, Faculty of Pharmacy, CEU San Pablo University, Madrid, Spain; 2 Spanish Nutrition Foundation (FEN), Madrid, Spain; 3 Department of Biochemistry and Molecular Biology II and Institute of Nutrition and Food Sciences, University of Granada, Granada, Spain; 4 Department of Preventive Medicine and Public Health, University of Navarra, Pamplona, Spain; 5 CIBEROBN, Biomedical Research Networking Center for Physiopathology of Obesity and Nutrition, Carlos III Health Institute, Madrid, Spain; 6 ImFINE Research Group, Department of Health and Human Performance, Technical University of Madrid, Madrid, Spain; 7 Department of Nutrition, Faculty of Pharmacy, Madrid Complutense University, Madrid, Spain; 8 Research Institute of Biomedical and Health Sciences, University of Las Palmas de Gran Canaria, Las Palmas de Gran Canaria, Spain; Universitat de Lleida-IRBLLEIDA, SPAIN

## Abstract

**Background:**

Folates and vitamin B_12_ are key nutrients in one-carbon metabolism and related diseases. Updated and plausible information on population intakes and their major dietary sources is scarce and urgently needed in Spain in order to increase the knowledge that can lead as previous step to prevention by fortification and supplementation policies.

**Aims:**

The present study aims to evaluate main dietary folate and vitamin B_12_ sources and intakes in the Spanish population.

**Materials and methods:**

Results were derived from the ANIBES cross-sectional study using a nationally representative sample of the Spanish population (9–75 years, n = 2,009).

**Results:**

Food groups with the highest mean proportional contribution to total folate intakes in both males and females were vegetables (21.7–24.9%) and cereals (10.7–11.2%), while meat and meat products (26.4%) and milk and dairy products (27.3%) were for B_12_. Total median folate and B_12_ intakes amongst women were 156.3 μg/d and 4.0 μg/d while for men were 163.6 μg/d and 4.5 μg/d, respectively. In all age groups, vitamin intakes were significantly higher in plausible than in non-plausible energy reporters.

**Conclusion:**

A limited number of participants had adequate folate intakes, whereas vitamin B_12_ intakes were adequate for practically the entire population. There is a clear need for improving folates intake in the Spanish population.

## Introduction

Folic acid and vitamin B_12_ are two metabolically and clinically linked vitamins, which share some key functions related to the one-carbon metabolism [1]. At present, the main research challenges are related to the study of folate and B_12_ interactions and the dietary adequacy of vulnerable population groups. Since 1940, folic acid (FA) has been used for the prevention and treatment of macrocytic or megaloblastic anaemia [2]. From the 1990s, new potential functions were described for the vitamin, being the prevention of congenital malformations such as neural tube defects (NTDs), the most demonstrated [3]. Other new functions are the well-established regulation of homocysteine concentrations (a cardiovascular risk factor) [1], the prevention or promotion of colorectal cancer depending on timeframe [4] and the maintenance or improvement of cognitive function in seniors [5]. Also important to mention, is its role in immune function [6] and more recently on osteoporosis prevention [7] and hearing loss [8]. Folates are the generic name given to the natural FA vitamers found in vegetable and some animal food products. Vitamin B_12_, also known as cobalamin is also an essential molecule for humans. It acts as a cofactor in one-carbon transfers through methylation and molecular rearrangement. The most frequent clinical expression of vitamin B_12_ deficiency is megaloblastic anaemia; and it has also been associated with many neurological disorders, although neurological signs might appear earlier than haematological [9, 10]. Moreover, the most well-known adverse effect of supplementation and food fortification with FA is the masking of the diagnosis of vitamin B_12_ deficiency, because megaloblastic anaemia caused by cobalamin deficiency can be reversed, unlike the potential detrimental long-term neurological effects [11–13].

Prevalence of marginal folate and vitamin B_12_ deficiency in western countries is increasing [14–16], but no parallel concern in society and public health policies has been observed. In Europe, it was shown that sub-clinical deficiency of folates and vitamin B_6_ could affect around 20% of European adolescents [17]. Likewise, the analysis of nutrient intake data from a review of a number of European countries showed a higher risk of inadequate folate intakes in adults and the elderly population/seniors when compared to the rest of the population [18]. Equally, the information of the European Nutrition and Health Report I provides an overview of folates inadequacy in European seniors [19]. Furthermore, Planells et al. [20] have given a precise estimate of the nutritional status for vitamins B_6_, B_12_ and folates in the adult population of southern Spain, and they have shown that factors such as age, place of residence, level of education and smoking habits can increase the risk of inadequate intake of these nutrients.

From 1998, the United States of America (USA) and Canada started the implementation of a nutrition policy of mandatory fortification with FA in flour and derived grain products; today mandatory fortification is present in up to 80 countries, but none of them European [21]. This nutrition policy was established following a proposal by the Food and Drug Administration aimed at preventing NTDs [22], and figures have indeed shown that prevalence rates of infants born with NTDs have successfully decreased in the USA as well as in other countries [23]. In Spain, where no mandatory fortification policy exists, an important number of food products, such as breakfast cereals and milk products are voluntarily fortified with FA [24]. Noteworthy, it has been shown in these products that overages are a current practice as analysed FA values were, in most cases, higher than those declared in nutritional labels by manufacturers [25]. In our country, it seems that this practice by the food industry is not improving the nutritional status of the population: Planells et al. [20] observed that the percentage of individuals with an acceptable vitamin status (>6 ng/ml) reached 57.6% for folates and 89.1% for vitamin B_12_ (>200 pg/ml). However, in the Spanish National Survey of Dietary Intake (Encuesta Nacional de Ingesta Dietética en España, ENIDE) [26] vitamin B_12_ intakes were higher than national reference intakes, ranging from 300% to 400% of the Recommended Dietary Intakes (RDI), remarkably different from the observed intakes of FA, ranging from 59% to 77%, regardless of gender and age.

At present, the assessment of dietary intakes of a population is determined by nutritional surveys whose main limitation is the indirect, inaccurate nature of the method and frequently, reported data do not represent the habitual intake of the studied population. Therefore, nutritional surveys estimate energy intakes (EI) that might not be physiologically plausible [27]. In this regard, the European Food Safety Authority (EFSA) [28] has published a protocol with a harmonized approach to identify misreporting based on a review of the methods used in representative samples of people aged 10–74 years in Europe, and suggests that the data should be reported for the whole population as well as divided into plausible and non-plausible energy reporters. In addition, the literature shows that methods that use innovative information and communication technologies to collect dietary information may improve the quality and accuracy of studies [29] as it is widely accepted that completing food records may be burdensome for survey participants.

For all above-mentioned, urges the need of an improved and updated knowledge of the micronutrient intakes in the Spanish population to prevent and/or delay the adverse effects resulting from inadequate intakes at different stages of life. The ANIBES study is the first national dietary survey in Spain to implement novel collection methods for comprehensive dietary data. In addition, it is the only representative study of the Spanish population that used new technologies such as tablet devices to record food intakes and leftovers. Hence, in the present study, the aims were to examine the contribution of different food groups and subgroups and to acknowledge the dietary intakes of folates and vitamin B_12_ in the Spanish population according to age, gender and misreporting.

## Materials and methods

The complete design, protocol and methodology of the ANIBES study ("Anthropometry, Intake and Energy Balance in Spain") have been described in detail elsewhere [30–32] and references are fully available at the repository from the Spanish Nutrition Foundation (FEN) http://www.fen.org.es/anibes/es/biblioteca.

### Sample

The ANIBES study is a cross-sectional study conducted using stratified multistage sampling. To guarantee better coverage and representativeness, the fieldwork was performed at 128 sampling points across Spain. The design of the ANIBES study aims to define a sample size that is representative of all individuals living in Spain, aged 9–75 years, and living in municipalities of at least 2,000 inhabitants. The initial potential sample consisted of 2,634 individuals, and the final sample comprised 2,009 individuals (1013 men, 50.4%; 996 women, 49.6%). In addition, for the youngest age groups (9–12, 13–17, and 18–24 years), an “boost sample” was included to provide at least n = 200 per age group (error ± 6.9%). The augment sample is the process of increasing the amount of interviews for a particular subgroup within the population in order to achieve an adequate number of interviews to allow analysis of population subgroups or segments that wouldn't normally yield a sufficient number of interviews in a main random survey, without the expense of increasing the sample size for the whole survey. Therefore, the random sample plus boost sample comprised 2,285 participants. The sample quotas according to the following variables were: age groups (9–12, 13–17, 18–64, and 65–75 years), gender (male/female), geographical distribution (Northeast, Levant, Southwest, North–Central, Barcelona, Madrid, Balearic, and Canary Islands) and locality size: 2000 to 30,000 inhabitants (rural population): 30,000 to 200,000 inhabitants (semi-urban population) and over 200,000 inhabitants (urban population). Additionally, other factors for sample adjustment were considered: unemployment rate, percentage of foreigners (immigrant population), physical activity level and education or economic level. The fieldwork for the ANIBES study was conducted from mid-September 2013 to mid-November 2013, and two previous pilot studies were also performed. To equally represent all days of the week, study subjects participated during two weekdays and one weekend day. The final protocol was approved by the Ethical Committee for Clinical Research of the Region of Madrid, Spain [31].

### Food and beverage records and adequacy of intakes

Study participants were provided a tablet device (Samsung Galaxy Tab 2 7.0, Samsung Electronics, Suwon, South Korea) and trained in recording information by taking photos of all food and drinks consumed during the 3 days of the study, both at home and outside. Different days of the week would be equally represented as each 3 day cycle included two working days (Monday and Tuesday or Thursday and Friday), and one weekend day (Saturday for Thursday and Friday cycle or Sunday for Monday and Tuesday cycle). Photos had to be taken before and after each eating and drinking occasion, in order to record the actual intake. Additionally, a brief description of meals, recipes, food brands, and other relevant information was recorded using the tablet. Participants who declared that were unable to use the device were offered other options, such as using a digital camera and paper record and/or telephone interviews. A total 79% of the sample used a tablet, 12% a digital camera, and 9% opted for a telephone interview. As no differences in the percentage of misreporting were found according to the type of device used to assess dietary intake, we used the measurements of the three assessment methods in the analysis. Participants provided detailed information regarding each eating and drinking occasion including not only what and how much was eaten, but also where and with whom they shared their meals, if they were watching television and/or sitting at a table. In addition, they were asked to declare if their intake was representative for that specific day (e.g. in case of a medical condition such as gastroenteritis that might compromise dietary habits) and to record any dietary supplement taken. The survey also contained a series of questions about participants’ customary eating habits (e.g., the type of milk usually consumed) to facilitate further coding. Food records were returned from the field in real time, to be coded by trained personal who were supervised by dieticians. An *ad hoc* central server software/database was developed for this purpose, to work in parallel with the codification and verification processes. This software/database was developed to obtain information from field tablets every 2 seconds, and the database was updated every 30 minutes. Data obtained from food manufacturers and nutritional information provided on food labels were also included. A photographic food atlas was used to assist in assigning gram weights to portion sizes. Food, beverage, energy and nutrient intakes were calculated from food consumption records using the VD-FEN 2.1 software, a Dietary Evaluation Program from the Spanish Nutrition Foundation (FEN), Spain, which was newly developed for the ANIBES study based mainly on the Spanish Food Composition Tables [33], with several expansions and updates. Energy distribution and objectives for the Spanish population were used to analyse the overall quality of the diet [31, 34]. Micronutrient reference intakes for Spain were used to compare the actual reported intake with those recommended [35]. The adequacy of folate and B_12_ intakes were then expressed as the percentage of population achieving >80% of the Recommended Dietary Intakes (% above 80% RDI) for each vitamin according to Moreiras et al. [35].

### Evaluation of misreporting

National diet and nutritional surveys are the most used tools to assess diet, nutrient intake and nutritional status of the population. Data collected in these surveys is mostly based on subject self-reporting. As this method is indirect and has an inaccurate nature, surveys frequently report data that do not represent the habitual intake of the studied population and estimates energy intakes (EI) that might not be physiologically plausible [27]. EFSA recommendations were followed to calculate misreporting; in which the proposed protocol is based on Goldberg [36] and Black [37]. This method evaluates the reported EI (EIrep) against the presumed energy requirements. EIrep is expressed as a multiple of the mean basal metabolic rate estimate (BMRest), and it is compared with the presumed energy expenditure of the studied population. Then, the ratio EIrep:BMRest is referred to as the physical activity levels (PAL) [28]. Detailed methodology has been detailed in previous publications from the ANIBES study [38].

### Statistical analysis

Results are expressed as median (interquartile range) or as percentage. To establish if the samples were parametric or non- parametric, the Kolmogorov–Smirnov test was used. The non-parametric data were statistically analysed by the Kruskal–Wallis test. When Kruskal–Wallis test resulted in differences, multiple comparisons between medians were studied by Mann-Whitney’s U test. Differences were considered significant at p<0.05. Data analysis was performed with SPSS 22.0 software package (IBM Corp., Armonk, NY, USA).

## Results

The final ANIBES study population included 996 females and 1,013 males (Table 1). In the entire population, the plausible energy reporters were 543 individuals (27%), and the non plausible energy reporters were 1,466 (73%). A higher proportion of non-plausible energy reporters were identified amongst males (53.3%, Table 1). In addition, 75.9% adults were acknowledged as non plausible energy reporters. In our study, the lowest proportion of non-plausible energy reporters was observed in children (5.8%), adolescents (8.4%) and seniors (10.0%) (Table 2).

**Table 1:** Description of the ANIBES sample by gender and reporting.

Gender	Reporting	
	Plausible energy reporters	Non-plausible energy reporters
**Female**	57.3% (n = 311)	46.7% (n = 685)
**Male**	42.7% (n = 232)	53.3% (n = 781)

**Table 2:** Description of the ANIBES sample by gender, reporting and age group.

		Children (9–12 y)	Adolescents (13–17 y)	Adults (18–64 y)	Seniors (65–75 y)
**Gender**	**Female**	7.7% (n = 87)	6.6% (n = 74)	76.2% (n = 857)	9.5% (n = 107)
	**Male**	10.9% (n = 126)	11.8% (n = 137)	68.8% (n = 137)	8.5% (n = 137)
**Reporting**	**Plausible energy reporters**	17.8% (n = 120)	11.3% (n = 76)	64.2% (n = 433)	6.7% (n = 45)
	**Non-plausible energy reporters**	5.8% (n = 93)	8.4% (n = 135)	75.9% (n = 1222)	10.0% (n = 161)

Total median folates and vitamin B_12_ intakes amongst females were 156.3 μg/d and 4.0 μg/d respectively while for males were 163.6 μg/d and 4.5 μg/d (Tables 3 and 4). Significantly higher intake values (p<0.001) were observed in the plausible energy reporters group in both females and males either for folates and vitamin B_12_: female’s daily folates intakes were 185.1 μg/d and vitamin B_12_ 4.6 μg/d, whereas for males were 205.8 μg/d and 5.7 μg/d of folates and B_12_, respectively. Potential prevalence of adequacy for folates and vitamin B_12_ (% population above 80% RDI) in the total study population according to the national criteria, the Recommended Dietary Intakes by Moreiras et al. [35], is also presented by gender and reporting in Tables 3 and 4. The proportion of adequacy for folates in total women population was 3.0% and 6.6% for men (Table 3). When considering plausible energy reporters, women’s proportion of adequacy increased to 5.5% and men’s to 14.2%. Vitamin B_12_ adequacy in total female and male population accounted for 93.4% and 96.6%, respectively. When only plausible energy reporters were studied, these proportions increased to 97.4% and 99.6% in females and males respectively.

**Table 3:** Folate intakes (μg/d) and prevalence of adequacy (percentage of population above 80% RDI) in ANIBES sample by gender and reporting.

Gender	Folates (μg/d)	% above 80% RDI
**Females**		
Total n = 996	156.3 (116.2–207.1)	3.0
Plausible energy reporters n = 331	185.1*** (143.7–237.4)	5.5
Non-plausible energy reporters n = 685	141. 0 (105.8–183.9)	1.9
**Males**		
Total n = 1013	163.6 (125.6–213.3)	6.6
Plausible energy reporters n = 232	205.8*** (165.3–269.5)	14.2
Non-plausible energy reporters n = 781	151.7 (116.9–195.0)	4.4

RDI: Recommended Dietary Intakes [36]. Values are median (interquartile range) per group.

*** p<0.001 difference plausible vs non-plausible energy reporters (Mann-Whitney’s U test).

**Table 4:** Vitamin B_12_ intake (μg/d) and prevalence of adequacy (percentage of population above 80% RDI) in ANIBES sample by gender and reporting.

Gender	Vitamin B_12_ (μg/d)	% above 80% RDI
**Females**		
Total n = 996	4.0 (2.7–5.6)	93.4
Plausible energy reporters n = 331	4.6*** (3.4–6.5)	97.4
Non-plausible energy reporters n = 685	3 (2.4–5.1)	91.5
**Males**		
Total n = 1013	4.5 (3.3–6.2)	96.6
Plausible energy reporters n = 232	5.7*** (4.3–7.9)	99.6
Non-plausible energy reporters n = 781	4.2 (3.0–5.8)	95.8

RDI: Recommended Dietary Intakes [36]. Values are median (interquartile range) per group.

*** p<0.001 difference plausible vs non-plausible energy reporters (Mann-Whitney’s U test).

Tables 5 and 6 show the results of the analysis by age group and reporting. Total median folate intakes were higher in adults (160.3 μg/d) and seniors (163.0 μg/d) while the lowest values were observed in adolescents (154.6 μg/d). Total median B_12_ intakes were higher in children (4.1 μg/d) and adults (4.3 μg/d) while lower in seniors (3.8 μg/d) and adolescents (3.9 μg/d). Vitamin intake values in plausible energy reporters were significantly higher (p<0.001) than in non-plausible energy reporters in all age-groups. Noteworthy, folate intakes were higher in the senior plausible energy reporters group (227.3 μg/d) and B_12_ in the plausible energy reporter’s adults and senior group (5.3 μg/d). Conversely, children’s plausible energy reporters group presented lower folates (173.7 μg/d) whereas children’s and adolescent’s plausible energy reporters group had lower B_12_ (4.5 μg/d) median daily intakes.

**Table 5:** Folates intake (μg/d) and prevalence of adequacy (percentage of population above 80% RDI) in ANIBES sample by age group and reporting.

Age Group	Folates (μg/d)	% above 80% RDI
**Children**		
Total n = 213	156.0 (124.1–197.5)	24.9
Plausible energy reporters n = 120	173.7*** (135.7–210.9)	31.7
Non-plausible energy reporters n = 93	138.3 (101.2–176.8)	16.2
**Adolescents**		
Total n = 211	154.6 (113.7–208.9)	3.8
Plausible energy reporters n = 76	203.4*** (144.0–241.4)	9.2
Non-plausible energy reporters n = 135	139.3 (99.5–179.7)	0.8
**Adults**		
Total n = 1655	160.3 (119. 6–209.3)	4.1
Plausible energy reporters n = 433	201.9*** (156.8–252.7)	7.2
Non-plausible energy reporters n = 1222	146.1 (110.5–189.6)	3.0
**Seniors**		
Total n = 206	163.0 (124.2–228.7)	4.9
Plausible energy reporters n = 45	227.3*** (178.2–272.1)	11.1
Non-plausible energy reporters n = 161	152.7 (114.8–208.5)	3.1

RDI: Recommended Dietary Intakes [36]. Values are median (interquartile range) per group.

*** p<0.001 difference No misreporting vs Misreporting (Mann-Whitney’s U test)

**Table 6:** Vitamin B_12_ intake (μg/d) and prevalence of adequacy (percentage of population above 80% RDI) in ANIBES sample by age group and reporting.

Age Group	Vitamin B_12_ (μg/d)	% above 80% RDI
**Children**		
Total n = 213	4.1 (3.0–5.6)	91.2
Plausible energy reporters n = 120	4.5*** (3.6–6.4)	100.0
Non-plausible energy reporters n = 93	3.3 (2.6–5.1)	93.5
**Adolescents**		
Total n = 211	3.9 (2.8–5.5)	94.8
Plausible energy reporters n = 76	4.5*** (3.4–6.2)	97.4
Non-plausible energy reporters n = 135	3.5 (2.7–4.9)	93.3
**Adults**		
Total n = 1655	4.3 (3.0–6.0)	94.9
Plausible energy reporters n = 433	5.3*** (3.9–7.2)	97.7
Non-plausible energy reporters n = 1222	4.0 (2.7–5.6)	93.9
**Seniors**		
Total n = 206	3.8 (2.9–5.8)	95.2
Plausible energy reporters n = 45	5.3*** (3.5–7.9)	100.0
Non-plausible energy reporters n = 161	3.6 (2.7–5.3)	93.8

RDI: Recommended Dietary Intakes [36]. Values are median (interquartile range) per group.

*** p<0.001 difference No misreporting vs Misreporting (Mann-Whitney’s U test)

When folate and vitamin B_12_ intakes were assessed by geographical distribution, overall North Central, Barcelona (metropolitan area) and Northeast regions presented higher folate intakes: 180.1 μg/d, 179.9 μg/d and 162.7μg/d, respectively (Table 7), while the lowest folate intakes were observed in southern Spain. Therefore, there were significantly higher folate intakes in the Northeast (p<0.01), North Central (p<0.001) and Barcelona (p<0.001) regarding observed South peninsular areas. There were also significantly lower folate intakes in Madrid, Levante and the peninsular northwest (p<0.05) comparing to intakes of the population of Barcelona. Higher vitamin B_12_ intakes were also reported in the North Central region (4.9 μg/d). Significantly higher vitamin B_12_ intakes were also observed in the North Central region comparing to Madrid (p<0.05), Northeast (p<0.01), Southern Iberian Peninsula (p<0.001) and the Canary Islands (p<0.01).

**Table 7:** Folates and vitamin B_12_ intake (μg/d) by geographical distribution.

Geographical distribution (Nielsen Areas)	Folates (μg/d)	Vitamin B_12_ (μg/d)
Barcelona (metropolitan Area)	179.8*** (140.0–241.2)	4.2 (3.0–6.1)
Canary Islands	159.9 (130.3–216.8)	4.5^▲▲^ (2.7–5.4)
Center	160.9 (121.0–222.4)	4.4 (3.1–5.9)
Levante	160.8^#^ (118.3–208.0)	4.4 (3.1–6.1)
Madrid (Metropolitan Area)	156.4^#^ (126.1–201.9)	4.2^▲^ (2.9–5.7)
Northeast	162.7** (125.8–230.1)	4.1^▲▲^ (2.7–5.8)
Northwest	163.5 ^#^ (112.2–207.4)	4.3 (2.9–6.1)
North Central	180.1*** (133.8–226.9)	4.9 (3.6–7.3)
South	149.8 (109.6–196.4)	4.2^▲▲^ (2.8–5.9)

Values are median (interquartile range) per group.

** p<0.01 difference vs South (Mann-Whitney’s U test)

*** p<0.001 difference vs South (Mann-Whitney’s U test)

^#^ p<0.05 difference vs Barcelona (Mann-Whitney’s U test)

^▲^ p<0.05 difference vs North Central (Mann-Whitney’s U test)

^▲▲^ p<0.01 difference vs North Central (Mann-Whitney’s U test)

### Contribution of dietary food sources to folate and vitamin B_12_ intakes

**Fig 1:**
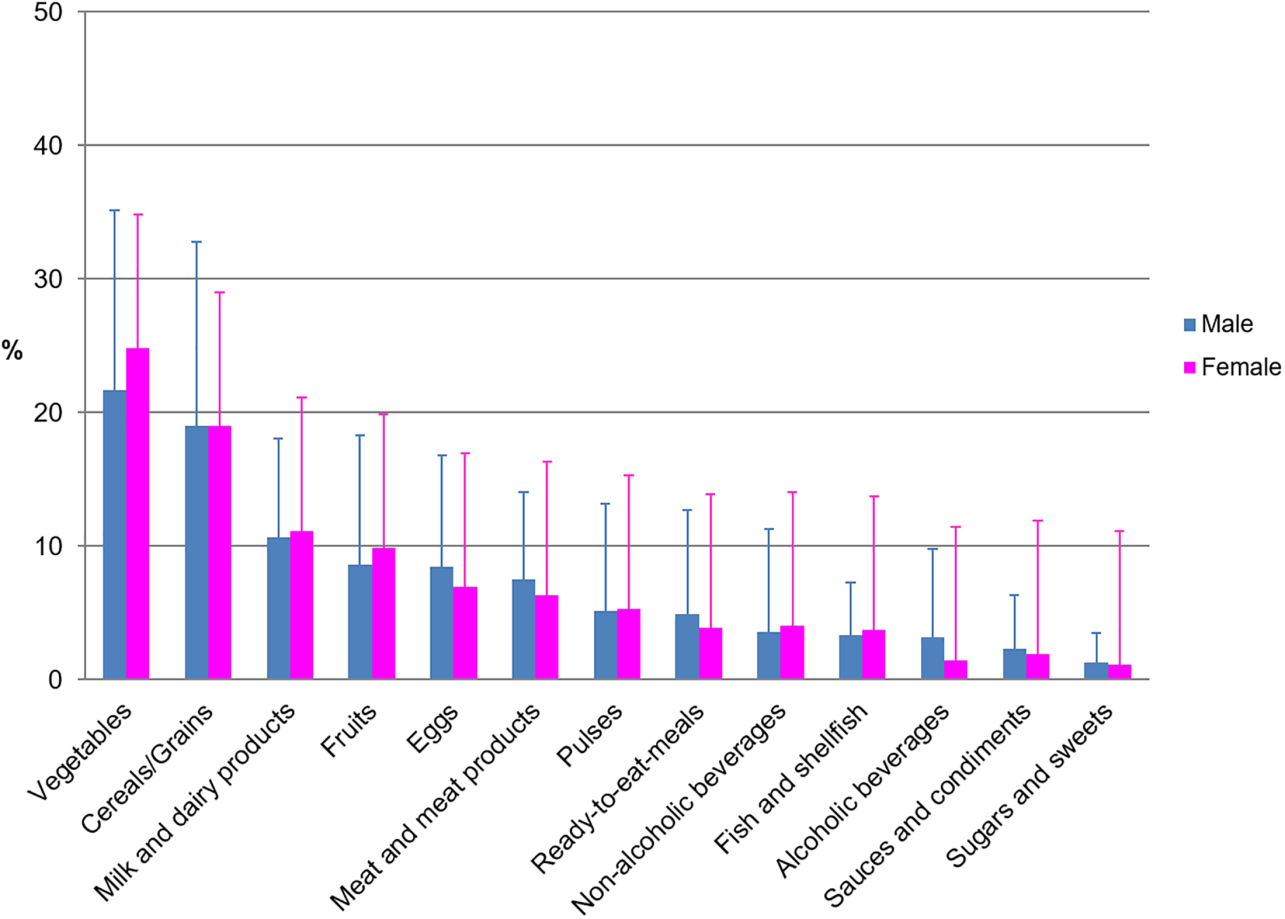
Contribution of food and beverages to folates intakes by gender.

**Fig 2:**
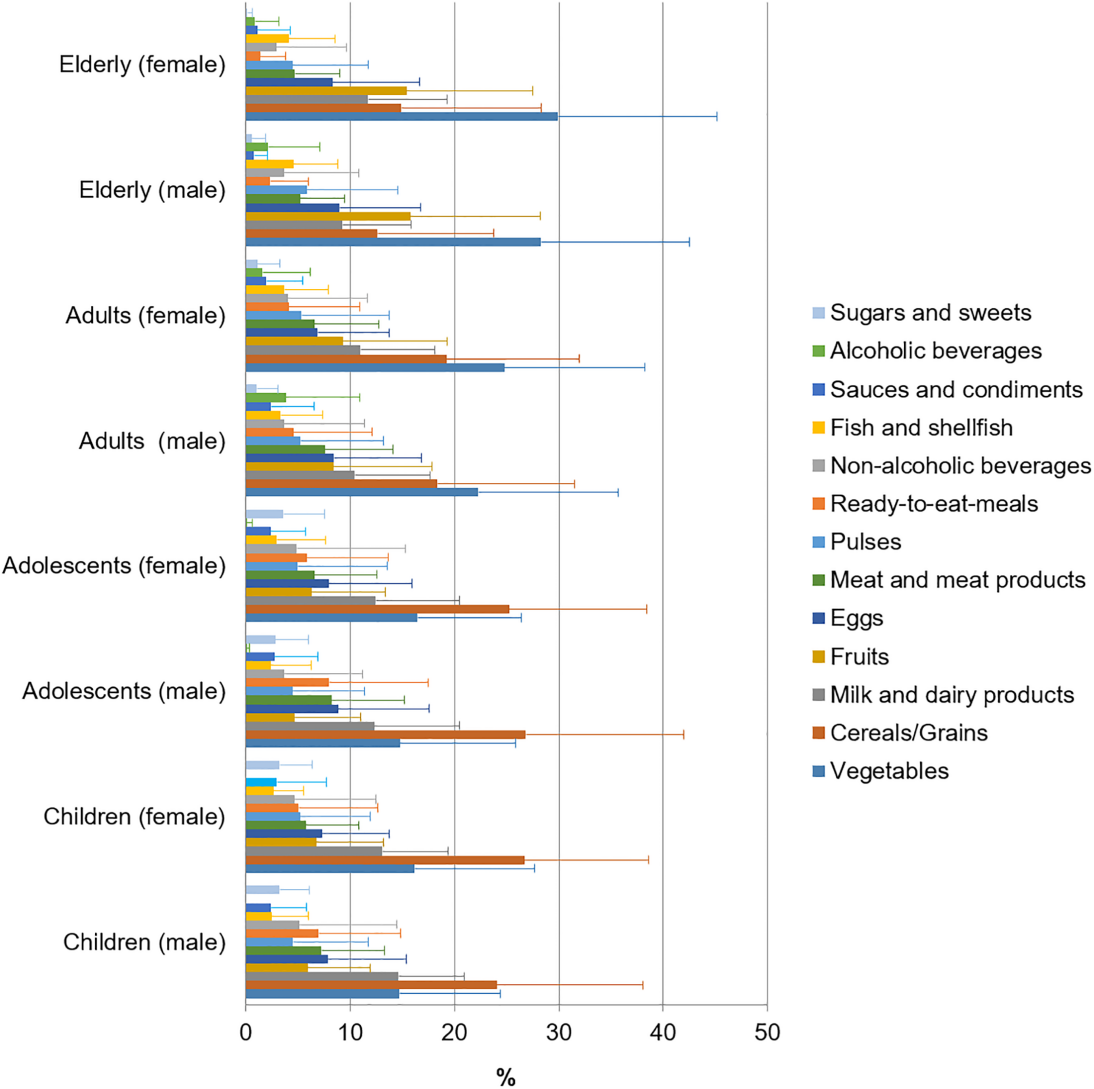
Contribution of food and beverages to folate intakes by gender and age group.

The contribution of food and beverage groups (%) to the daily folates intake is shown in Fig 1, where differences between gender are included. The food groups with the highest mean proportional contribution to total folate intake in both males and females were vegetables (21.7–24.9%) and cereals (10.7–11.2%). It is worth highlighting that in Fig 1, only those foods which contribute at least 1% to folate intakes of the population have been included. In contrast, when participants were compared according to age group (Fig 2), we found that in children and adolescents, cereals, vegetables and milk and dairy products supplied 25.7%, 15.5% and 13.1% of folate intakes, respectively. However, for adults and seniors, vegetables, cereals and milk and dairy products supplied the largest percentage of intakes: 26.3%, 16.3% and 10.6%, respectively. Remarkably, legumes and pulses only made a minor contribution regardless of age-group (4.5–5.8%) being lowest in male adolescents and higher in senior men.

**Fig 3:**
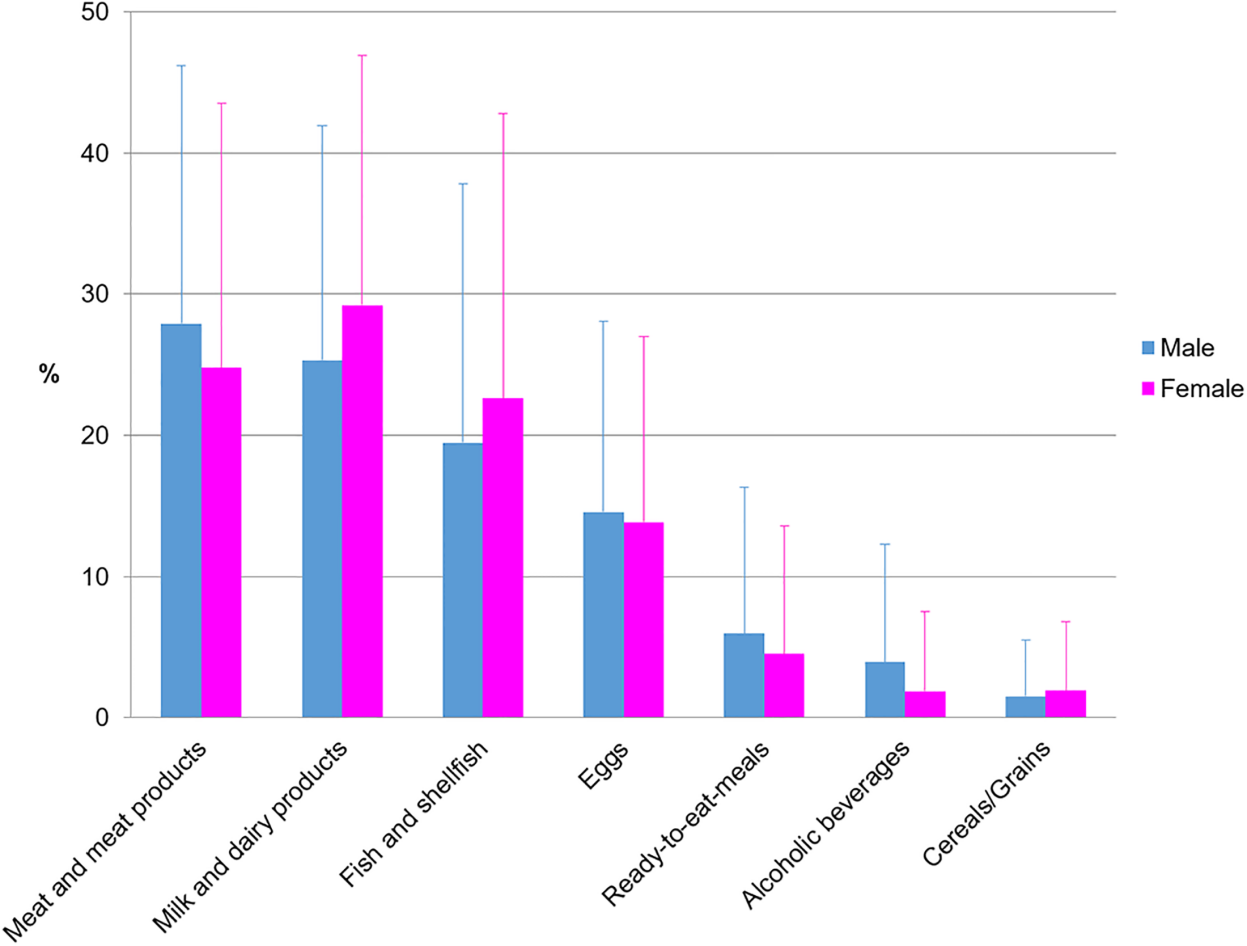
Contribution of food and beverages to vitamin B_12_ intakes by gender.

Meat and meat products (27.9%), milk and dairy products (25.3%) and fish and shellfish (19.4%) were the main sources of vitamin B_12_ for men while in the case of females, milk and dairy products (29.2%) were the greatest contributors, followed by meat and meat products (24.8%) and fish and shellfish (22.6%) (Fig 3).

**Fig 4:**
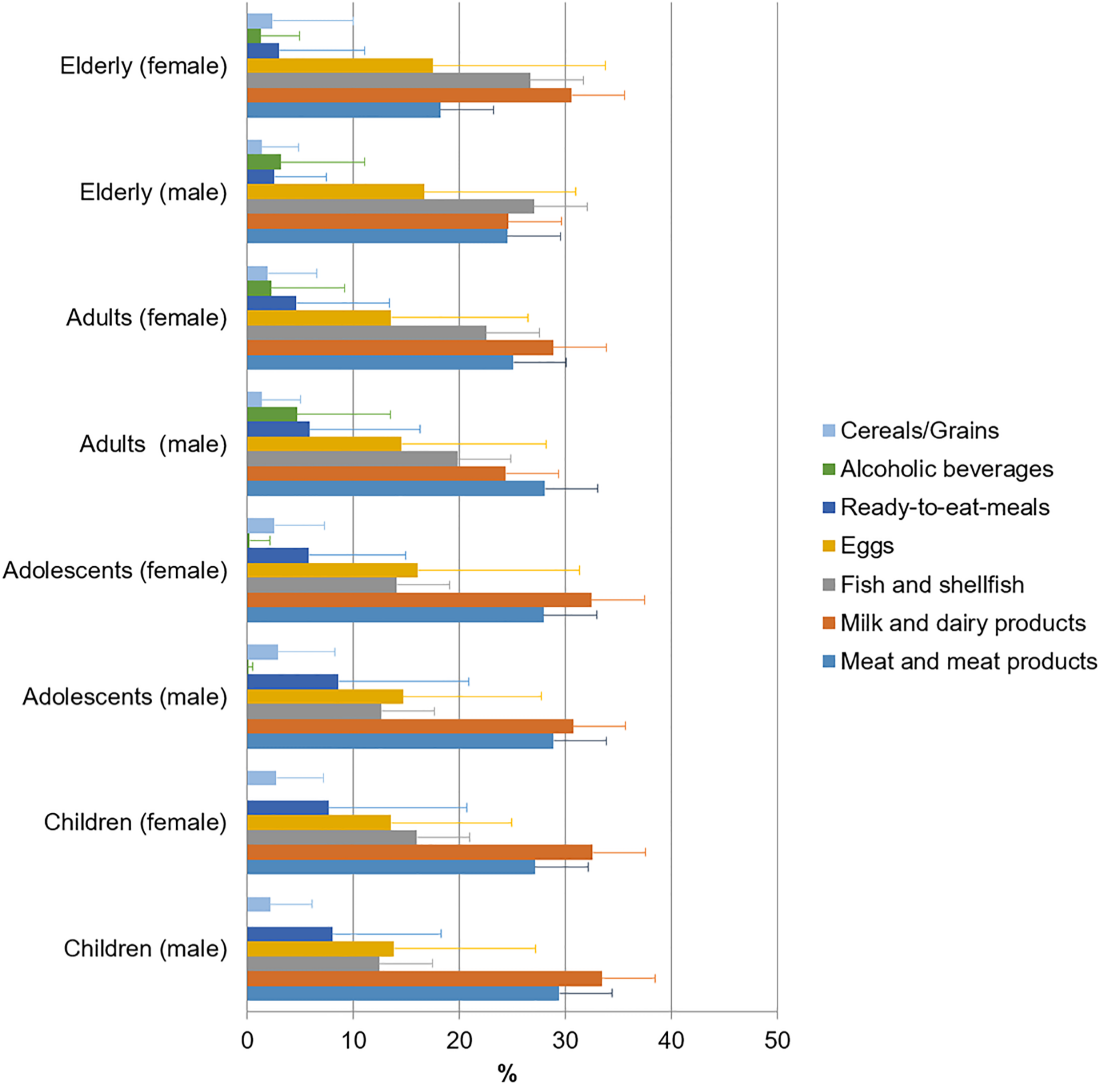
Contribution of food and beverages to vitamin B_12_ intakes by gender and age group.

When studying the main sources of vitamin B_12_ for each age-population group (Fig 4), we observed that for children and adolescents, both males and females, milk and dairy products were the highest contributors (30.7–33.4%) followed by meat and meat products (27.1–29.4%). Male adults consumed higher proportions of meat and meat products than females (28.0 vs. 25.0%). The target age-population group for vitamin B_12_ intakes are seniors, and in our study, we found that the main vitamin source for senior men were fish and shellfish (27.0%) in contrast to women where milk and dairy products contributed to a higher proportion of intakes (30.5%).

## Discussion

The purpose of the present pioneer study was to evaluate dietary intakes of folate and vitamin B_12_ from the ANIBES population according to age, gender and misreporting, by using new technologies to record food and beverage consumption, as well as to examine the major food sources of both vitamins. It is acknowledged that in Europe the prevalence of sub-clinical deficiency for folates and vitamin B_12_ could affect around 20% of European adolescents [17]. Likewise, a study analysing the nutrient intake data in Europe showed a higher risk of inadequate FA intakes for adults and senior population [18]. This emerging concern is of particular importance in vulnerable age-groups, such as children, women of childbearing age and seniors. In this regard, the primary finding of our study is that very few people from the ANIBES population had adequate folate intakes, while virtually the entire population had adequate vitamin B_12_ intakes. Our data are in line with those collected in the Healthy Lifestyle in Europe by Nutrition in Adolescence (HELENA) study [39], in which it was observed that folate intakes were not achieved in older children in other European countries. In this age group, it is worth recalling the special importance of folates during periods of rapid cell division and growth [40]. On the other hand, hyperhomocysteinemia (HHcy) has been related to memory loss and the development of dementia in the elderly. HHcy and low dietary folate are being considered as potential independent risk factors for the development of Alzheimer’s disease and other neurodegenerative dementias [41]. Also, HHcy is an independent risk factor for memory deficit and the development of cognitive impairment without dementia [42]. It has been hypothesised that the association between Hcy and cognitive performance in aging may be attributed to the exposure to Hcy neurotoxic effects [43–46] together with folate, vitamin B_6_ and B_12_ deficiencies [47–49].

Remarkably, intakes for both vitamins, in male and female groups, were higher in reported data that might not represent the usual intake of the studied population. Indeed, underreporting in the present study has the same pattern for both vitamins regardless of the population group that we study. Our results were higher when compared to those published in 2009 by Tabacchi et al. [50] in a revision concerning the adequacy of micronutrient intake in Europe. Authors observed that folates inadequacy across eight countries encompassed about 25% of the adult female population, as they had inadequate intakes when assessed according to a number of folate recommendations selected by the investigators. However, nearly 75% of these women had inadequate intakes when evaluated using the folate Estimated Average Requirement cut-point value of 320 μg/d. Therefore, it is feasible to acknowledge that results comprising adequacy of micronutrients largely depend on the Dietary Recommendation reference of choice [50, 51]. Moreover, it must be taken into account when establishing dietary reference intakes, that most studies recognize women and older subjects as frequent non-plausible energy reporters, but also very importantly, those with a higher body mass index [52] and then, may be selective for different kinds of foods and nutrients [53, 54]. Our results indicate that almost half of the respondents (46.7% of women and 53.3% of men) were non plausible energy reporters, and that adults were the largest group of non-plausible energy reporters (75.9%) followed by seniors (10.0%), adolescents (8.4%) and children (5.8%). This prevalence is consistent with the one observed in other international studies in which it is shown that the misreporting of energy intake in nutrition surveys is widespread [27, 55–63], although, only a few studies have examined the misreporting in children and adolescents [64–69].

A wide range of plants but only limited animal foods are natural sources of folates: green leafy vegetables, liver, yeast, legumes and pulses, being the most significant [70]. Our findings show that vegetables, milk and dairy products and fruits are the main dietary sources of folates found in the ANIBES study. However, other results as those obtained in the ENIDE dietary survey in Spain [26] showed that a higher percentage of folates contribution were supplied by legumes and seeds (30%), with vegetables and cereals being second (28%) and third (12%), respectively. Planells et al. [20] observed higher contributions from fruits (25.5%) and lower from milk and cheese to total folates intake. These differences may be explained in part due to the age difference of the total sample in each of the mentioned studies: in the study carried out by Planells et al. [20] the population were between 25 and 60 y old, in the ENIDE dietary survey in Spain [26] the population were between 18 and 64 y old, while in the ANIBES study population were aged 9 to 75 years. Therefore, when we analyse the sample according to the age group, we observed that cereals, vegetables, milk, and dairy products are the food groups that largely contribute to folate intakes in children and adolescents. However, for adults and seniors, vegetables, cereals and milk and dairy products were the main sources. Legumes and pulses are a rich folate source, but according to our results, this food group is amongst the lowest folate contributors to the Spanish diet. In this regard, recent publications from the ANIBES study [31] and by Varela-Moreiras et al. [71] have confirmed the declining intakes of this staple food from the Mediterranean diet. Contrary to the Spanish population, in other countries, especially where mandatory fortification is implemented, such as Brazil, the major contributors of folate intake are processed foods made from wheat flour fortified with FA [72]. It is worth mentioning that folate intakes from the ANIBES study were assessed only with food composition data from natural food sources. Voluntarily fortified products were not identified and therefore folic acid intakes of the population sample could not be derived from our results. Some authors affirm that efforts to increase folate intake through natural folate-rich foods are unlikely to be effective for health prevention on a population basis and would leave a large portion of the population, particularly women of reproductive age, at increased risk for diseases related to folate insufficiency [73].

Vitamin B_12_ is exclusively found in animal-derived foods and only these natural sources are said to contain sufficient amounts to meet body cobalamin requirements [74]. Also, certain edible algae and fermented soybeans (tempe) contain some B_12_ but best dietary sources are ruminant meat and milk due to the natural bacterial populations that synthesize vitamin B_12_ in the rumen of these animals [74]. Our data are in accordance with that obtained in other European studies [20, 75], in which the proportion of vitamin B_12_ obtained from meat and dairy products was higher than in the USA [76], where cereals are a notable contributor to vitamin B_12_ intake in each ethnic-sex group. These variations could be attributed to the different geographical and cultural influences existing in the USA [77, 78]. Although according to our results vitamin B_12_ intakes are adequate for a high percentage of the ANIBES population, some specific population groups such as seniors and vegans cannot be ruled out as potentially having inadequate vitamin B_12_ intakes. In addition, latest Dietary Guidelines recommend decreasing the intake of meat and derivatives as the Spanish diet present an excessive amount of animal protein [34].

Worth mentioning, the strengths of the ANIBES study are the careful design, protocol, and methodology used and that it was performed in a representative random sample of the Spanish population aged 9–17 years. Furthermore, validated questionnaires used to collect information of food records have shown good reliability and reproducibility. A limitation of this study is its cross-sectional design, which provides evidence for associations but not for causal relationships. It is also important to underline that folate intakes were assessed with food composition data from natural food staples, recipes and products.

## Conclusions

In conclusion, our current results show that there is an important percentage of the Spanish ANIBES population not meeting the Recommended Intakes for folates while vitamin B_12_ consumption was adequate. The food sources that mostly contribute to folate intakes are vegetables, milk and dairy products and fruits, while the contribution to vitamin B_12_ intakes is mainly supplied from meat and meat products, milk and dairy products and fish and shellfish. The Spanish population would largely benefit from an increased ingestion of green-leafy vegetables, legumes, seeds and other uncooked plant-based foods, which will improve their folate intake and status. Additional research is needed to establish the best cost-effective public health approaches to achieve this goal.
